# Caspase 9 and Caspase 3 Immunohistochemical Pattern in Skeletal and Cardiac Muscles at Different Times after Death: An Experimental Study on PMI Estimation

**DOI:** 10.3390/diagnostics11061062

**Published:** 2021-06-09

**Authors:** Cristina Mondello, Chiara Stassi, Letteria Minutoli, Gennaro Baldino, Angela Alibrandi, Giovanni Francesco Spatola, Maria Laura Uzzo, Antonio Micali, Domenico Puzzolo, Alessio Asmundo, Elvira Ventura Spagnolo

**Affiliations:** 1 Department of Biomedical and Dental Sciences and Morphofunctional Imaging, University of Messina, Via Consolare Valeria, 1, 98125 Messina, Italy; modelloc@unime.it (C.M.); amicali@unime.it (A.M.); puzzolo@unime.it (D.P.); aasmundo@unime.it (A.A.); 2 Legal Medicine Section, Department for Health Promotion and Mother-Child Care, University of Palermo, Via del Vespro, 129, 90127 Palermo, Italy; chiara_stassi@libero.it (C.S.); gennarobld@hotmail.it (G.B.); 3 Department of Clinical and Experimental Medicine, University of Messina, Via Consolare Valeria, 1, 98125 Messina, Italy; lminutoli@unime.it; 4 Unit of Statistical and Mathematical Sciences, Department of Economics, University of Messina, Via dei Verdi 75, 98122 Messina, Italy; aalibrandi@unime.it; 5 Department of Biomedicine, Neurosciences and Advanced Diagnostics (BiND), University of Palermo, Via del Vespro, 129, 90127 Palermo, Italy; giovannifrancesco.spatola@unipa.it (G.F.S.); marialaura.uzzo@unipa.it (M.L.U.)

**Keywords:** caspase 3, caspase 9, skeletal muscle, cardiac muscle, time after death, post mortem interval, forensic sciences

## Abstract

(1) Background: The estimation of the post mortem interval (PMI) is a challenge for forensic pathologists because data emerging from methods commonly applied are not always conclusive, since several conditions exist that may affect the reliability of these parameters. Thus, new approaches have been proposed to overcome such a limit. In recent years, several studies have been performed on proteins analyzing their expression/degradation patterns in relation to the progressing of the post mortem interval. (2) Methods: The immunoreactivity patterns of two apoptosis mediators—Caspase 9 and Caspase 3—have been tested in order to evaluate their potential role as markers of the post mortem interval. The immunohistochemical analysis was performed on samples of skeletal and cardiac muscles obtained from rats at 0, 4, 8, 12, 24 and 72 h after death. (3) Results: The observed immunoreactivity patterns of both Caspase 9 and Caspase 3 showed a significant correlation with increasing post mortem interval either in skeletal or cardiac muscles, while the comparison of the immunoreactivity patterns of the two apoptotic mediators within each tissue appeared consistent with a preliminary activation of the “initiator” Caspase 9, which, in turn, subsequently activates the “executioner” Caspase 3. (4) Conclusion: The different expressions and decrease immunohistochemically observed on both caspases with progressing PMI support the usefulness of the combined analysis for post mortem interval estimation.

## 1. Introduction

The determination of the amount of time elapsed from one’s death to the recovery of the body—the so called post mortem interval (PMI)—has always relied on the integrated use of several methods, such as the temperature-based method of Hensgge, the electrical and mechanical excitability of the skeletal muscle, post mortem lividity development and rigor mortis progress [1,2,3,4,5]. Despite their remarkable value, such parameters allow to estimate only relatively short PMIs and within wide ranges; in addition, some of these methods are affected by several conditions—such as manner of death, particular environmental conditions and individual variations—which limit their practical application, proving not conclusive in terms of PMI estimation [6,7]. In fact, the Hensgge method is the only one that considers the cooling influencing conditions (i.e., body weight, mean ambient temperature, clothing/covering of the lower trunk, resting or moving air) providing corrective factors for a more objective and standardized estimation of PMI [1].

In order to overcome such a limit, many authors have engaged in the research of possible PMI markers by means of several approaches, such as biochemical analyses on body fluids and organs, flow cytometry, immunohistochemistry, metabolomics, RNA stability, and gene expression [6,7,8,9,10,11,12,13,14]. In this context, several proteins were analyzed in recent years with particular attention to the degradation patterns in different tissues over the time [15].

In this work, the immunoreactivity patterns of two apoptosis mediators—the “initiator” Caspase 9 and the “executioner” Caspase 3—have been evaluated with a semi-quantitative approach on tissue samples from 23 sacrificed rats obtained at different times after death; for their major stability over time, due both to the tissue characteristics and to their localization in more protected sites within the body, samples of the quadriceps femoris and left ventricular wall were chosen as matrices for the immunohistochemical assay. The aim of the experimental study was to evaluate if these apoptosis mediators might be used as markers for the estimation of early PMI; for this purpose, differences in immunopositivity observed in both tissues and time points after death were analyzed.

## 2. Materials and Methods

All procedures complied with the standards stated in the Guide for the Care and Use of Laboratory Animals (Institute of Laboratory Animal Resources, National Academy of Sciences, Bethesda, Maryland). Twenty-three male mice (25–30 g) were purchased from Charles River Laboratories Italia srl (Calco, LC, Italy). The animals were housed in plastic cages and fed a commercial pelleted diet within a humidity- and temperature-controlled room, with free access to water. All animals were sacrificed at the same time by an intraperitoneal overdose of ketamine and xylazine. The carcasses were assigned to six PMI groups (n. 4 to 0 h group, n. 4 to 4 h group, n. 4 to 8 h group, n. 3 to 12 h group, n. 4 to 24 h group, and n. 4 to 72 h group) and the bodies were stored at room temperature (24 ± 1 °C). Samples of the femoral quadriceps and left ventricular wall were collected at the designated time of PMI and fixed in 4% formalin.

All specimens fixed in 4% formalin were dehydrated in progressively increasing ethanol concentrations (from 75% to 100%), then clarified in xylene, and finally embedded in liquid paraffin at 65 °C. Ten slices 6 µm thick were obtained from each paraffin block using a Leica microtome RM2235, and then mounted on slides: one section was stained in hematoxylin-eosin in order to study the tissue morphology by light microscopy; the remaining nine sections were processed for the immunohistochemical assay and specifically, three sections were tested as negative controls, while Caspase 9 and Caspase 3 immunoreactivity was tested on three sections each.

### 2.1. Immunohistochemistry

The immunohistochemical assay was performed relying on a Mach 4 Universal HRP Polymer Detection System. Once set up, the slides were left to dry overnight at 37 °C and then stored at room temperature. The following day, the slides were dewaxed and rehydrated by sequential immersions in a graded series of alcohols (sequentially, xylene, ethanol 95% and ethanol 70%, for 5 min each), and then sequentially transferred into water for 5 min, into sodium citrate buffer pH 6 at 50 °C for 5 min and into PBS (Na_2_HPO_4_, KH_2_PO_4_, KCl, NaCl pH 7.4–7.6) for other 5 min. The endogenous peroxidase was inhibited by adding 3% hydrogen peroxide to the slides, and then incubating them for 5 min at room temperature in a humid atmosphere; subsequently, the slides were transferred into PBS at room temperature for 5 min, and then incubated overnight at 4 °C with monoclonal rabbit anti-Caspase 9 (Abcam, code ab222231) and monoclonal rabbit anti-Caspase 3 (Abcam, code ab224271) antibodies, both diluted to 1:100 in Da Vinci Green. Once the antibody excess was removed by rinsing the slides twice in PBS for 5 min each, Mach 4 HRP Polymer was added and incubations were run out for 30 min for those slides in which the anti-Caspase 9 primary antibody was placed, and for 15 min for those in which the anti-Caspase 3 primary antibody was placed. Betazoid DAB Chromogen diluted in Substrate Buffer was then added, and the colorimetric reaction was let running for 5 min before stopping it by rinsing the slides in distilled water. Finally, one drop of aqueous mounting medium, and a coverslip, were applied to each tissue section. Negative controls were obtained using PBS instead of the primary antibody (Supplementary Figure S1). For the positive control, mouse brain sections were used (Supplementary Figure S2).

### 2.2. Image Analysis

Each slide was examined using a Leica DMLB Microscope (Leica Microsystem GmbH Wetzlar, Germany), with a 10× field magnification. The images were captured by a Nikon DSfi1 Digital Image System and saved in a.TIFF format. Extended Adobe Photoshop CS6 (Adobe System Inc., San Jose, CA, USA) was used to elaborate the images and perform an image analysis. The images were analyzed with a CMYK color profile, using the yellow channel for a best linear response to color intensity. All images were converted to an inverted grey scale ranging from 0 to 255, and the mean colorimetric values were semi-quantitatively expressed as follows: “+” (1–50); “++” (51–100); “+++” (101–150); “++++” (151–200); “+++++” (201–255). Each sample was analyzed in double-blind by two different operators.

### 2.3. Statistical Analysis

Numerical data were expressed as mean values and standard deviation (SD), while categorical variables were expressed as number and percentage. A non-parametric approach was used, since the numerical variables were not normally distributed, as verified by the Kolmogorov–Smirnov test. The Friedman test was used to assess, at each time point, the immunoreactivity differences in Caspase 9 and Caspase 3 separately taken, both in skeletal and cardiac muscles (intra-group analysis). The casualization test was used to compare, for each tissue and at each time point, Caspase 9 and Caspase 3 immunoreactivities. Finally, the Mann–Whitney test was applied in order to evaluate the differences at each time point of both Caspase 9 and Caspase 3 immunohistochemical patterns between skeletal and cardiac muscles (inter-groups analysis). Statistical analyses were performed using IBM SPSS for Windows, v. 22 (IBM Corp., Armonk, NY, USA). A *p*-value smaller than 0.05 was considered statistically significant.

## 3. Results

### 3.1. Skeletal Muscle

**Figure 1:**
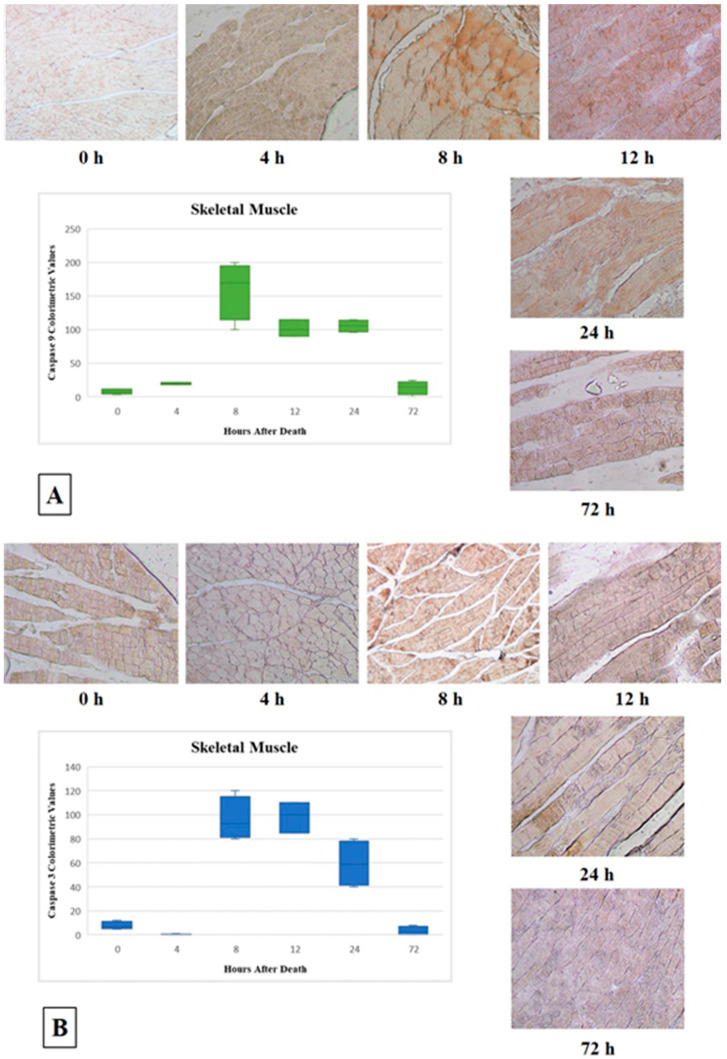
Graphs (boxplot) showing the median, minimum, and maximum of Caspase 9 and Caspase 3 expression (respectively (**A**,**B**)) in skeletal muscle at each PMI group and related representative images on immunopositivity observed at the designated time of PMI (Caspase 9—0 h: 10×; 4 h: 20×; 8 h: 10×; 12 h: 20×; 24 h: 20×; 72 h: 20×. Caspase 3—0 h: 10×; 4 h: 10×; 8 h: 10×; 12 h: 10×; 24 h: 20×; 72 h: 20×).

**Figure 2:**
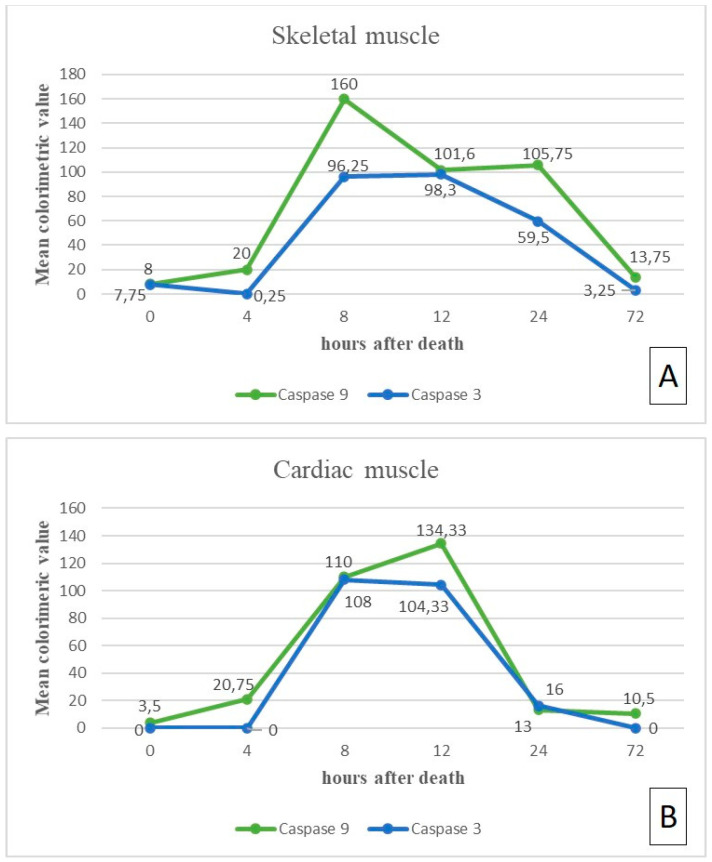
Graphs showing the medium value of immunopositivity observed at each time point for Caspase 9 and Caspase 3 respectively in skeletal muscle (**A**) and cardiac muscle (**B**): the skeletal muscle reactivity reveals an earlier pick of expression at 8 h, followed by a more gradual decrease of immunostaining, showing plateau stages at 8–12 h and 12–24 h respectively for Caspase 3 and Caspase 9; the reactivity in heart tissue shows an higher expression of Caspase 9 and 3 at 12 h, followed by an abrupt decrease and a plateau stage between 24 and 72 h.

The results of the immunohistochemical assay are reported in Table 1 and Figure 1 and Figure 2A. The immunoreactivity to both Caspase 9 and Caspase 3 resulted as negative when samples were obtained immediately after death (T_0_), with mean colorimetric values of 8 and 7.75, respectively. The samples obtained 4 h after death maintained a regular tissue architecture, showing a very low immunoreactivity to Caspase 9 (mean colorimetric value of 20) but no immunoreactivity at all to Caspase 3 (mean colorimetric value of 0.25). At 8 h after death, an increase in Caspase 9 immunoreactivity (mean colorimetric value of 160) and a modest Caspase 3 immunoreactivity (mean colorimetric value of 96.25) were observed. After 12 h, a diffuse alteration of the skeletal fibers’ morphology began to show, along with a diffuse, but decreased, immunoreactivity to Caspase 9 (mean colorimetric value of 101.66); no significant increase in Caspase 3 immunoreactivity was detected (mean colorimetric value of 98.33). The tissue morphology appeared further altered 24 h after death: in the remaining fibers, Caspase 9 immunoreactivity remained stable (mean colorimetric value of 105.75), while Caspase 3 immunoreactivity, although present, started decreasing (mean colorimetric value of 59.5). Finally, 72 h after death, the tissue architecture appeared completely lost, although showing in the remaining fibers a slight immunoreactivity to Caspase 9 (mean colorimetric value of 13.75); immunoreactivity to Caspase 3 was almost completely absent (mean colorimetric value of 3.25).

### 3.2. Cardiac Muscle

No immunoreactivity to both Caspase 9 and Caspase 3 was detected on any sample when obtained immediately after death (mean colorimetric value of 3.5 for Caspase 9; no signal for Caspase 3). At 4 h after death, the cardiomyocytes showed a very low immunoreactivity to Caspase 9 (mean colorimetric value of 20.75), but no immunoreactivity at all to Caspase 3. After 8 h, an immunoreactivity increase was detected for both Caspase 9 and Caspase 3 (mean colorimetric values of, respectively, 110 and 108.5). 12 h after death, the tissue architecture appeared only slightly modified; a diffuse, slight immunoreactivity increase to Caspase 9 (mean colorimetric value of 134.33) was observed, while Caspase 3 immunoreactivity remained stable (mean colorimetric value of 104.33). An increase in tissue morphology alteration was evident 24 h after death, together with a decrease of the immunoreactivity of both Caspase 9 and Caspase 3 (mean colorimetric values of, respectively, 13 and 16). Finally, a complete loss of the tissue morphology, along with a slight immunoreactivity to Caspase 9 (mean colorimetric value of 13), but not to Caspase 3 in the remaining cardiomyocytes, were observed 72 h after death.

**Figure 3:**
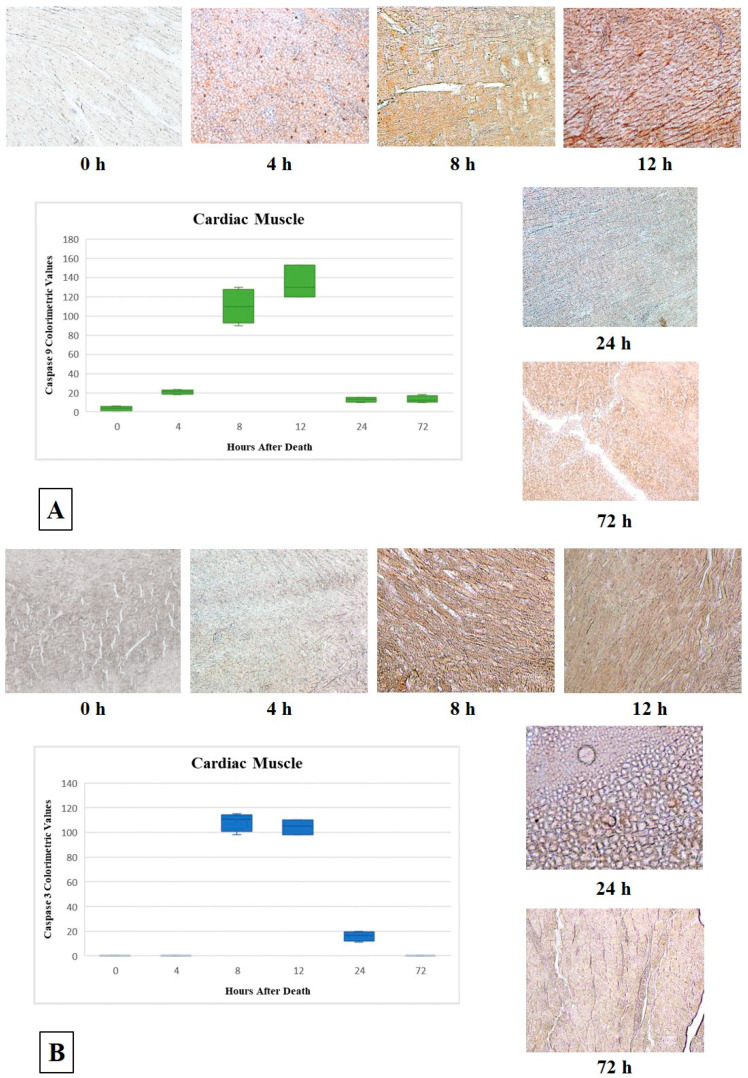
Graphs (boxplot) showing the median, minimum, and maximum of Caspase 9 and Caspase 3 expression (respectively (**A**,**B**)) in cardiac muscle at each PMI group and related representative images on immunopositivity observed at the designated time of PMI ((Caspase 9—0 h: 10×; 4 h: 10×; 8 h: 10×; 12 h: 10×; 24 h: 10×; 72 h: 10×. Caspase 3—0 h: 10×; 4 h: 10×; 8 h: 10×; 12 h: 10×; 24 h: 10×; 72 h: 10×).

The results of the immunohistochemical assay are reported in Table 1 and Figure 2B and Figure 3.

## 4. Discussion

Caspases are key components of the apoptotic pathway, being protease enzymes initiating and executing the process [16]. These molecules, in fact, are classified into initiator caspases (Caspase 2, 8, 9, 10) and executioner caspases (Caspase 3, 6, 7). Caspase 9 is an initiator with a caspase-recruitment domain, which is involved in the intrinsic mitochondrial pathway of apoptosis, which contributes to form apoptosomes [17]. Caspase 3 is an executioner activated by an initiator and involved in both apoptotic extrinsic and intrinsic pathways, which regulates the final apoptotic events, such as DNA fragmentation and plasmalemma blebbing [18].

Given their role in the apoptotic process, Caspase 9 and the Caspase 3 have been chosen to be immunohistochemically tested on samples of skeletal and cardiac muscles as possible markers for PMI determination. In fact, several studies described the use of semi-quantitative or quantitative methods for protein analysis on post-mortem human tissue samples to investigate PMI [15,19,20]. These researches, mainly, were focused on the progressive decrease of protein expression over the time after death to estimate the PMI, using several investigations, such as Western blotting, mass spectrometry and casein zymography [21,22].

The results of the presented study obtained on skeletal muscle showed an increasing trend of Caspase 9 immunoreactivity from 4 up to 8 h after death (*p* = 0.004), followed by stable—though slightly decreased—levels up to 24 h; a significant immunoreactivity decrease was observed 72 h after death. Caspase 3 immunoreactivity was absent up to 4 h and showed its highest level at 8 and 12 h after death (*p* = 0.002); a progressive decrease was observed at 24 and 72 h after death. The decreased levels of both caspases’ immunoreactivity at 24 and 72 h after death is in line with the appearance—at the same times—of increasing alterations of the tissue morphology at the hematoxylin-eosin stain. The comparison of Caspase 9 and Caspase 3 immunoreactivity patterns showed that, although Caspase 9 immunoreactivity tended to be slightly higher than that of Caspase 3 at each time point, the difference between the two apoptosis mediators was revealed to be statistically significant at 4 h (*p* = 0.046) (Figure 1 and Figure 2A). This is in line with the well-known apoptotic pathway, which sees a prior activation of the initiator caspases—such as Caspase 9—which, in turn, activate by proteolytic cleavage the executioner caspases—such as Caspase 3 [23,24].

A similar pattern, though with slight differences, was observed on samples of cardiac muscle. Caspase 9 immunoreactivity showed an increasing trend from 4 up to 12 h after death (*p* = 0.002), followed by an abrupt decrease at 24 and 72 h. As was observed on skeletal muscles, Caspase 3 immunoreactivity was absent on cardiac muscles up to 4 h, showing the highest signal at 8 and 12 h after death (*p* = 0.001); an abrupt decrease was observed at 24 and 72 h after death, in line with the on-set—at the same time points—of morphological alterations at the hematoxylin-eosin stain. Additionally, in this case, the comparison of Caspase 9 and Caspase 3 immunoreactivity patterns showed a statistically significant difference at 4 h after death (*p* = 0.046) (Figure 2B and Figure 3), thus confirming the temporal succession in Caspase 9 and Caspase 3 activation within the apoptotic pathway.

The immunohistochemical pattern of Caspase 9 in skeletal and cardiac muscles was revealed to be similar up to 8 h, whereas a significant higher signal was observed up to 12 h in cardiac muscles (*p* = 0.021). Relative to Caspase 3, the immunohistochemical pattern was similar between skeletal and cardiac muscles up to 12 h. Particularly, comparing the caspases’ expression between cardiac and skeletal muscles, some interesting findings were found. In heart tissue, even if a higher expression of Caspase 9 and 3 was observed at a later stage (12 h), an abrupt decrease was then observed characterized by a slight immunopositivity followed by a plateau stage between 24 and 72 h (Figure 2A). In skeletal muscle, the earlier peak of expression (at 8 h) was followed by a more gradual decrease of immunostaining showing plateau stages at 8–12 h and 12–24 h respectively for Caspase 3 and Caspase 9, and both proteins were still clearly detectable at 24 h (*p* = 0.020 for Caspase 9; *p* = 0.021 for Caspase 3) (Figure 2B). This evidence suggests that skeletal muscle could be a more suitable matrix in which the proteins degrade in a consistent manner with progressing PMI when compared to that detected in cardiac muscle. Several previous studies support the skeletal muscle suitability as a matrix to investigate for estimation of early and mild PMI with interesting and different findings on many proteins (i.e., troponin, desmin, vinculin, titin, calpain 1) [21,25,26].

To the best of our knowledge, no other work is present in literature that evaluates the Caspase 9 as a marker of PMI. Conversely, the results obtained for Caspase 3 are partly in line with Lee et al.’s work [27], where samples of rat kidneys and psoas muscles were used to evaluate at different times after death both the protein degradation and the immunohistochemical patterns of several possible PMI markers, included Caspase 3. Although no intermediate patterns between 0 and 24 h were evaluated, in line with our findings, also in Lee et al.’s study a progressive Caspase 3 decrease in psoas muscles was observed—both at Western blot and immunohistochemical assays—from 24 up to 72 h, when no Caspase 3 was present at all, thus leading the authors to consider Caspase 3 as a short-term marker of PMI as well.

Based on the present experimental study, it can be concluded that both Caspases 9 and 3 can be considered short-term markers for PMI determination. The different expressions and decreases, immunohistochemically observed on both proteins with progressing PMI, together with their functional (pathophysiological) correlation, support the usefulness of the combined analysis for PMI estimation. Obviously, these findings need the transposition from the experimental model to human material to provide a careful verification. In fact, other studies on this field revealed similar modification patterns of protein expression across species, supporting the usefulness of research on animal models. On the other hand, studies testing some proteins on both human and animal samples showed differences in protein degradation rates and the related timing [25,28,29].

A correct estimation of the PMI within the forensic field is of utmost importance since it does not provide just mere information, but also guides and complements criminal investigations in several contexts, such as in homicides. Since the existing methods are not always reliable, in recent years an increasing need for new approaches for PMI determination arose. To date, several methods have been proposed and tested, aiming to find one or more markers whose expression/activity/degradation patterns correlate with increasing times after death. Among all, the immunohistochemical approach has been chosen for the present work, since—in the perspective of a possible introduction as a routine assay—it represents an easy, fast and economic method, as well as being easily reproducible, resulting in widespread use in the forensic field [30,31,32]. Nonetheless, this being a pilot study, more investigations are needed to provide a careful validation for the application on samples obtained from human cadavers.

## Figures and Tables

**Table 1:** Immunohistochemical reactivity to Caspase 9 and Caspase 3 on skeletal and cardiac muscle samples at each time point after death.

Group	PMI	*N*	Skeletal Muscle				Cardiac Muscle			
			Caspase 9	Mean CV *	Caspase 3	Mean CV *	Caspase 9	Mean CV *	Caspase 3	Mean CV *
**G_1_**	*T*_0_ = 0 h	1	CV * 4 (+)	8 (+)	CV * 5 (+)	7.75 (+)	CV * 6 (+)	3.5 (+)	0	0
		2	CV * 6 (+)		CV * 8 (+)		CV * 4 (+)		0	
		3	CV * 12 (+)		CV * 6 (+)		CV * 4 (+)		0	
		4	CV * 10 (+)		CV * 12 (+)		0		0	
**G_2_**	*T*_1_ = 4 h	5	CV * 20 (+)	20 (+)	0	0.25 (+)	CV * 22 (+)	20.75 (+)	0	0
		6	CV * 22 (+)		CV * 1 (+)		CV * 18 (+)		0	
		7	CV * 18 (+)		0		CV * 23 (+)		0	
		8	CV * 20 (+)		0		CV * 20 (+)		0	
**G_3_**	*T*_2_ = 8 h	9	CV * 200 (+ + + +)	160 (+ + + +)	CV * 120 (+ + +)	96.25 (+ +)	CV * 100 (+ +)	110 (+ + +)	CV * 110 (+ + +)	108.5 (+ + +)
		10	CV * 160 (+ + + +)		CV * 80 (+ +)		CV * 130 (+ + +)		CV * 115 (+ + +)	
		11	CV * 180 (+ + + +)		CV * 85 (+ +)		CV * 90 (+ +)		CV * 98 (+ +)	
		12	CV * 100 (+ +)		CV * 100 (+ +)		CV * 120 (+ + +)		CV * 111 (+ + +)	
**G_4_**	*T*_3_ = 12 h	13	CV * 115 (+ + +)	101.66 (+ + +)	CV * 100 (+ +)	98.33 (+ +)	CV * 120 (+ + +)	134.33 (+ + +)	CV * 110 (+ + +)	104.33 (+ + +)
		14	CV * 100 (+ +)		CV * 110 (+ + +)		CV * 153 (+ + + +)		CV * 98 (+ +)	
		15	CV * 90 (+ +)		CV * 85 (+ +)		CV * 130 (+ + +)		CV * 105 (+ + +)	
**G_5_**	*T*_4_ = 24 h	16	CV * 100 (+ +)	105.75 (+ + +)	CV * 80 (+ +)	59.5 (+ +)	CV * 10 (+)	13 (+)	CV * 15 (+)	16 (+)
		17	CV * 112 (+ + +)		CV * 73 (+ +)		CV * 12 (+)		CV * 18 (+)	
		18	CV * 96 (+ +)		CV * 45 (+)		CV * 15 (+)		CV * 20 (+)	
		19	CV * 115 (+ + +)		CV * 40 (+)		CV * 15 (+)		CV * 11 (+)	
**G_6_**	*T*_5_ = 72 h	20	0	13.75 (+)	0	3.25 (+)	CV * 10 (+)	13 (+)	0	0
		21	CV * 15 (+)		0		CV * 18 (+)		0	
		22	CV * 15 (+)		CV * 8 (+)		CV * 12 (+)		0	
		23	CV * 25 (+)		CV * 5 (+)		CV * 12 (+)		0	

* CV: Colorimetric Value.

## Data Availability

All data supporting the reported results can be found in the manuscript and Supplementary Materials.

## Supplementary Materials

Positive and negative control images are available online at https://www.mdpi.com/article/10.3390/diagnostics11061062/s1. Figure S1. Negative control, Figure S2. Positive control.
