# Supplementary material for: Caspase 9 and Caspase 3 Immunohistochemical Pattern in Skeletal and Cardiac Muscles at Different Times after Death: An Experimental Study on PMI Estimation
Source: Diagnostics (Basel). 2021 Jun 9;11(6):1062. doi: 10.3390/diagnostics11061062 (PMC8229155; doi:10.3390/diagnostics11061062)
Supplement: Supplementary file 1 [file diagnostics-11-01062-s001.zip › diagnostics-1247134-SI.pdf]

Supplementary material

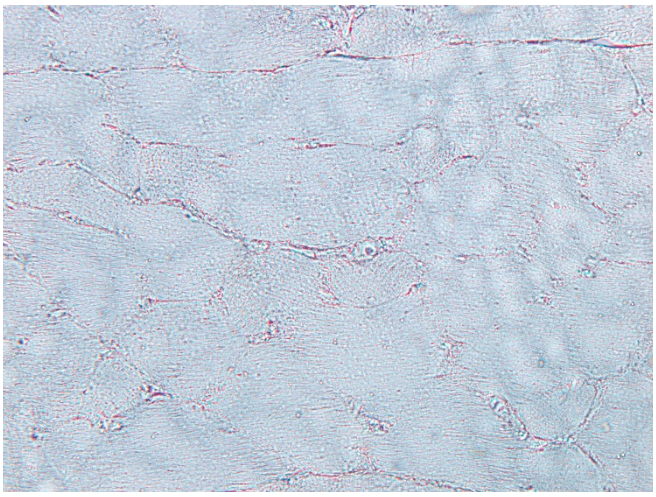

Supplementary Figure S1. Representative image of negative controls obtained, using PBS, from skeletal muscle (10×).

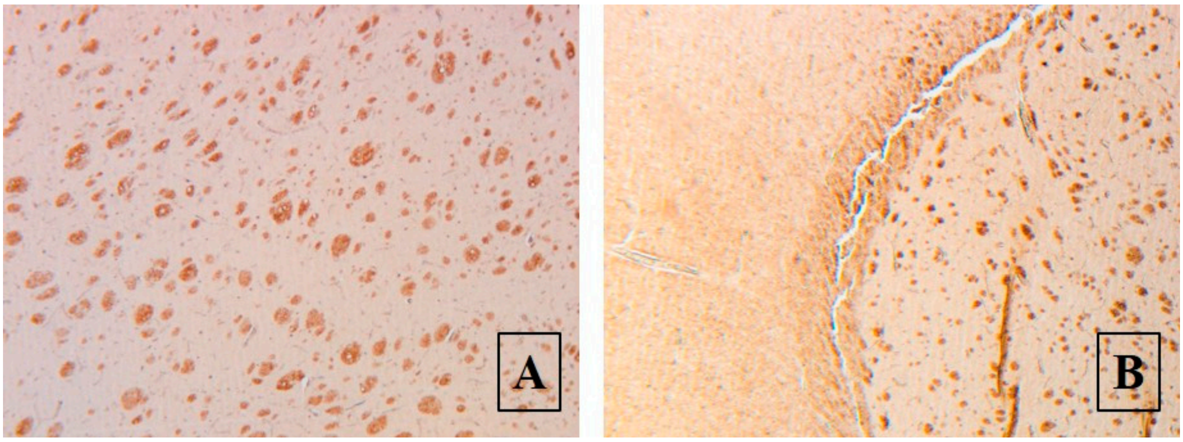

Supplementary Figure S2. Representative image of mice brain section used as positive control (Caspase 9 (**A**: 10×); Caspase 3 (**B**: 10×)).
